# Jejunal obstruction due to jejunocolic congenital band in a 12-year-old child: a case report

**DOI:** 10.1186/s13256-022-03546-w

**Published:** 2022-11-10

**Authors:** Gulan Maree, Ali Alelayan, Ferhad Hemi, Waseem Shater, Alaa Ghuzlan, Wajih Ali

**Affiliations:** Pediatric Surgery Department, Tishreen University Hospital, Lattakia, Syria

**Keywords:** Congenital band, Intestinal obstruction, Jejunum

## Abstract

**Background:**

A congenital band is an uncommon abnormality that can be found anywhere along the gastrointestinal tract. Intestinal obstruction caused by an anomalous congenital band is very rare in children. To the best of our knowledge, no cases of congenital bands extending from the descending colon to the jejunum have been reported in the English literature

**Case presentation:**

Herein, we present the case of a 12-year-old Syrian patient with intestinal obstruction due to a congenital band extending from the mesentery of the descending colon to the mesentery of the jejunum with an entrapped loop of jejunum between the band and the mesentery. The location of the obstruction was determined by upper gastrointestinal contrast radiography, but the cause of the obstruction was diagnosed intraoperatively. The band was excised without intestinal resection.

**Conclusion:**

Prediagnosis of congenital bands can be challenging, and surgery is required. When making a bowel obstruction differential diagnosis, it is important to keep this type of band in mind.

## Background

Small bowel obstruction (SBO) caused by an abnormal congenital band is extremely rare in children [1]. Obstruction occurs when the small bowel is compressed by thes  bands or when small bowel loops become trapped between the band and the mesentery [2]. It is extremely difficult to establish a preoperative diagnosis of a congenital band [3]. Congenital bands that cause obstruction must be treated surgically [4]. To the best of our knowledge, only about 50 cases of congenital bands in children have been reported in the medical literature, and none of these cases involved congenital bands that extend from the descending colon to the jejunum [5].

## Case presentation

**Fig. 1:**
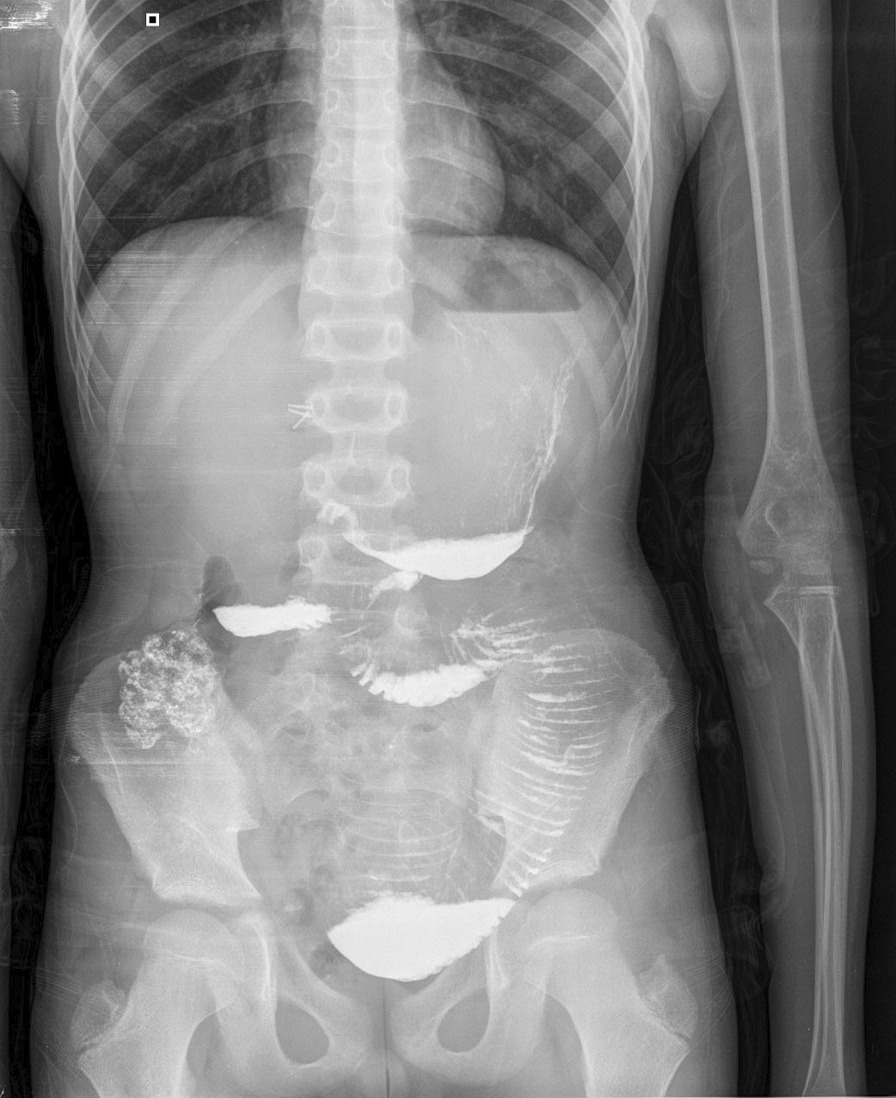
Contrast study showing dilated bowels at the level of the jejunum

**Fig. 2:**
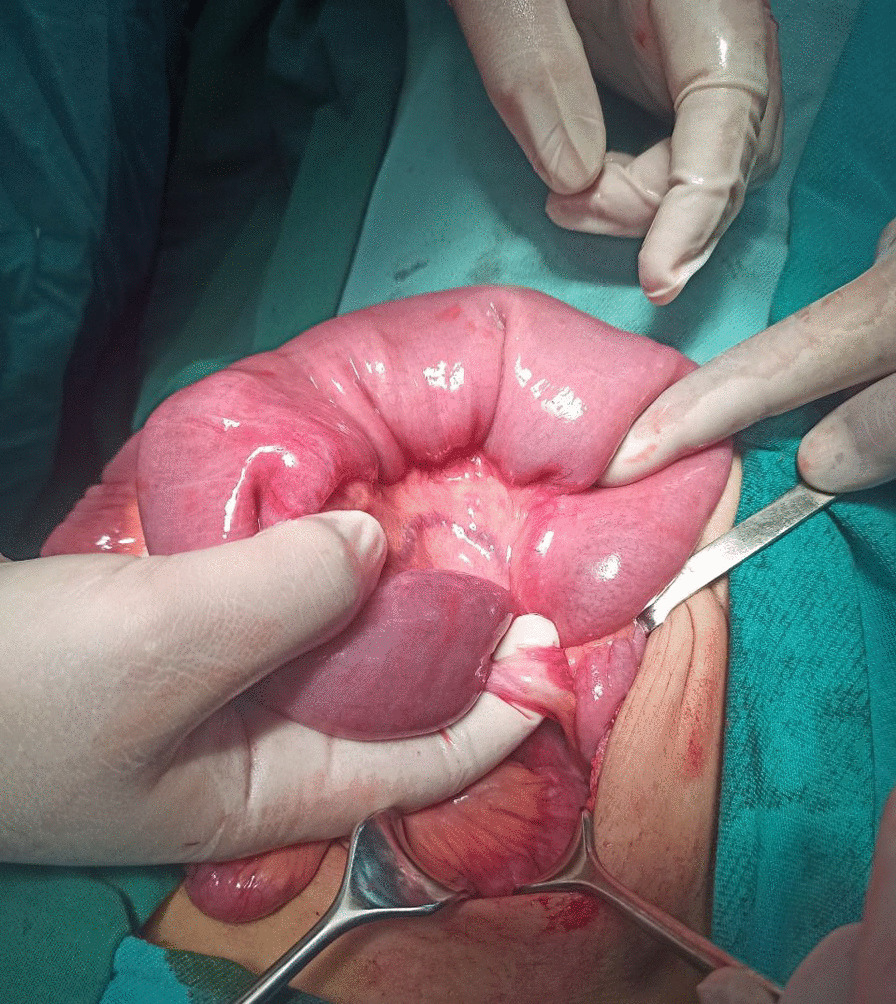
Image of an internal hernia of a section of the jejunum without signs of perforation

**Fig. 3:**
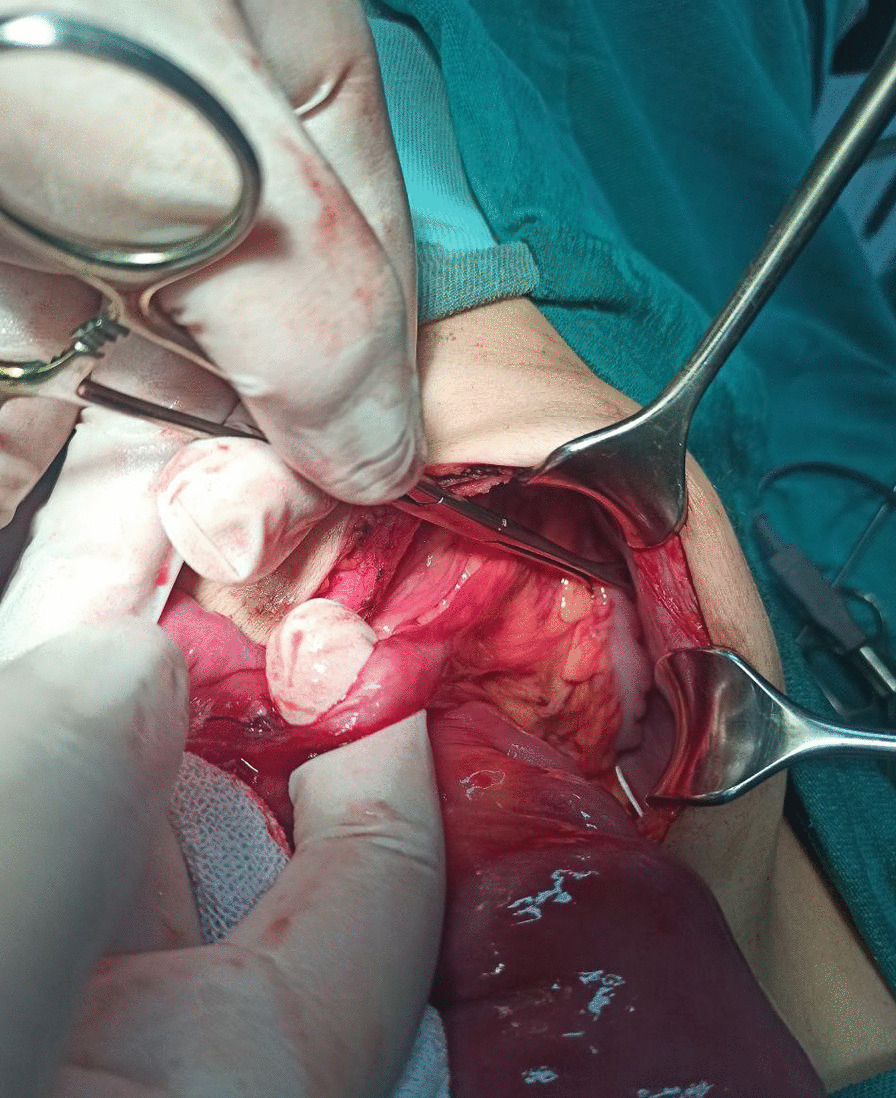
Image of a band extending from the colonic mesentery to the jejunal mesentery

A 12-year-old Syrian boy was transferred to our hospital with complaints of recurrent bilious vomiting, constipation, and crampy abdominal pain for 6 days. His physical examination showed abdominal distention and tenderness. The vital signs were stable, and there was mild dehydration. All laboratory results were normal. Plain X-rays revealed a dilated bowel with air–fluid levels. An upper gastrointestinal contrast study showed dilated bowels in the upper part of the abdomen, including the stomach, duodenum, and jejunum (Fig. 1). Intravenous fluids were administered, and nasogastric tubes were placed. On laparotomy, a congenital band was found connecting the mesentery of the descending colon to the mesentery of the jejunum, with an entrapped loop of jejunum between the band and the mesentery (Fig. 2). The appearance of the intestines was normal with normal blood supply. This band was ligated and excised without bowel resection (Fig. 3). Histological examination revealed fibrotic tissue with blood vessels and no malignant changes. The patient was discharged on the 4th postoperative day, and no complications occurred during the 10-month follow-up period.

## Discussion

In children, intestinal bands caused by inflammation and surgery are the most common causes of intestinal obstruction [6]. Congenital bands are uncommon in children, with the ileum being the most common location, followed by the colon mesentery, omentum, peritoneum, jejunum, and every other site of the gastrointestinal tract, including the stomach [7]. Various obstruction sites have been reported in the literature, with the ileum being a common location. Akgur *et al*. described eight cases of bands, the most common of which were between the right colon and ileum, as well as the Treitz angle and the terminal ileum, the right lobe of the liver and the ileum, and the right lobe of the liver and the right colon [6]. There have been two other rare cases reported, one involving the iliac fossa and the sigmoid mesocolon and the other involving the jejunum and the jejunum [8, 9]. Kerkeni *et al*. reported ten patients ranging in age from 1 day to 9 years. The most common anatomic location of congenital bands (five patients) was jejunum–jejunum [5]. Basak Erginel *et al*. reviewed the records of 14 children aged 4 days to 12 years who had intestinal obstructions caused by anomalous congenital bands. In four cases, congenital bands were found between the ascending colon and the mesentery of the terminal ileum [10]. The child in our case had no prior history of abdominal surgery, trauma, infection, inflammatory diseases, or peritonitis. The congenital band was found between the mesentery of the jejunum and the mesentery of the colon, which is an extremely rare occurrence, especially at this age, when most cases occur at a younger age. Internal hernia caused an obstruction at the level of the jejunum, approximately 60 cm from the Treitz ligament. The cause of congenital band is unknown, but it is not caused by known embryologic remnants such as the omphalomesenteric duct or vitelline vessel remnants [11].

Although radiologic examinations such as abdominal radiography or computed tomography scans can help differentiate between diseases, they are ineffective in making a correct preoperative diagnosis of intestinal obstruction caused by a congenital band.

Once a congenital band is suspected, it should be determined whether malrotation exists [1].

In our case, the radiographic evaluation did not assist us in diagnosing this condition. On contrast, the location of the intestinal obstruction was determined, and some other causes of obstruction were ruled out by the upper gastrointestinal contrast study. The appearance of the intestine was normal, with no malrotation. Surgical treatment is essential in the management of a congenital band. Laparotomy has traditionally been recommended, but with the advent of minimally invasive surgery, laparoscopy has been proposed as an alternative. Wu *et al*. recently reported that laparoscopy in the diagnosis and treatment of a congenital band may be safe and feasible [4]. The decision to perform a laparotomy was made on the basis of the patient’s symptoms of recurring vomiting, abdominal pain, and constipation, as well as the radiographic findings. We did not perform a laparoscopy because it was not available at our hospital.

## Conclusion

A congenital band is a rare malformation that can exist anywhere along the gastrointestinal tract. It is extremely rare to see these bands between the colonic and jejunal mesenteries. Prediagnosis can be challenging, and surgery is necessary to avoid further complications. When making a bowel obstruction differential diagnosis, it is important to keep this type of band in mind.

## Data Availability

All data generated or analyzed during this study are included in this published article.
